# Variations in the Regulatory Region of Alpha S1-Casein Milk Protein Gene among Tropically Adapted Indian Native (*Bos Indicus*) Cattle

**DOI:** 10.5402/2013/926025

**Published:** 2013-01-14

**Authors:** Amit Kishore, Manishi Mukesh, Ranbir C. Sobti, Bishnu P. Mishra, Monika Sodhi

**Affiliations:** ^1^Cattle Genomics Laboratory, National Bureau of Animal Genetic Resources, P.O. Box 129, Karnal, Haryana 132001, India; ^2^Department of Biotechnology, Panjab University, Chandigarh 160014, India; ^3^Buffalo Genomics Laboratory, National Bureau of Animal Genetic Resources, P.O. Box 129, Karnal, Haryana 132001, India

## Abstract

Regulatory region of milk protein alpha S1-casein (*αS1*-*CN*) gene was sequenced, characterized, and analyzed to detect variations among 13 Indian cattle (Bos indicus) breeds. Comparative analysis of 1,587 bp region comprising promoter (1,418 bp), exon-I (53 bp), and partial intron-I (116 bp) revealed 35 nucleotide substitutions (32 within promoter region, 1 in exon-I, and 2 in partial intron-I region) and 4 Indels. Within promoter, 15 variations at positions −1399 (A > G), −1288 (G > A), −1259 (T > C), −1158 (T > C), −1016 (A > T), −941 (T > G), −778 (C > T), −610 (G > A), −536 (A > G), −521 (A > G), −330 (A > C), −214 (A > G), −205 (A > T), −206 (C > A), and −175 (A > G) were located within the potential transcription factor binding sites (TFBSs), namely, NF-*κ*E1/c-Myc, GATA-1, GATA-1/NF-E, Oct-1/POU3F2, MEF-2/YY1, GATA-1, AP-1, POU1F1a/GR, TMF, GAL4, YY1/Oct-1, HNF-1, GRalpha/AR, GRalpha/AR, and AP-1, respectively. Seventy-four percent (26/35) of the observed SNPs were novel to Indian cattle and 11 of these novel SNPs were located within one or more TFBSs. Collectively, these might influence the binding affinity towards their respective nuclear TFs thus modulating the level of transcripts in milk and affecting overall protein composition. The study provides information on several distinct variations across indicine and taurine *αS1*-*CN* regulatory domains.

## 1. Introduction

 Bovine caseins are distinguished into four protein fractions, namely, alpha S1-casein, alpha S2-casein, beta-casein, and kappa-casein encoded by genes: *α*S1-CN, *α*S2-CN, *β-CN, *and *κ-CN*, respectively [1]. Alpha S1-casein represents the major protein fraction (31%) among the bovine milk proteins (caseins and whey) and constitutes up to 40% of total casein [1]. It is a calcium sensitive and highly phosphorylated protein. It has an important role in the capacity of milk to transport calcium phosphate and is organized at 5′-terminus of casein cluster located on bovine chromosome 6 (BTA6). Till now, 9 variants (A-I) have been reported in the coding region of *αS1-CN*. Amongst these, B and C, differing in amino acid substitution (Glu/Gly) at position 192 of the mature protein, are the most common. The variant C has been reported to be common in zebu breeds, while other rare variants like A, D, and F have only been reported in European cattle [2]. These variants are well characterized and their associations with quantitative effects on milk performance/production parameters have been widely reported [3, 4]. The results on association studies involving only coding region variants are not always consistent [5] and this might be attributed to the presence of intragenic haplotypic combination of variants in the regulatory and coding regions [3, 6]. Moreover, casein gene expression is also known to be differentially regulated by hormones and most of the potential hormone receptor binding sites occur within the 5′-flanking region of casein genes [6]. Thus, mutations at these regulatory regions might also have enduring effect on milk protein gene regulation at transcriptional level [6, 7] either individually or as inter- or intragenic haplotypes. For *αS1-CN*, mutations in the promoter region have been reported to influence the protein-coding efficiency of the relevant structural gene by changing the binding affinity towards their respective nuclear transcription factors (TFs) [8, 9] and can thus be considered as functional candidate for milk protein content. Additionally, variants of *αS1-CN5*′-flanking region ( *αS1-CN5*′) have also been associated with economically important traits like longevity and somatic cell scores in different taurine breeds [3, 10, 11]. Sequence variation within *αS1-CN5*′ has been widely studied in several species like cattle involving mainly *B. taurus* [10–14], yak [15], buffalo [16], goat [17], and sheep [18]. In contrast to the studies conducted on *αS1-CN5*′ in *B. taurus* and few zebu cattle [12, 19], no systematic study has been made to reveal the variations and haplotypes existing in *αS1-CN5*′ among Indian cattle breeds. The Indian native (*B. indicus*) breeds are adapted to diverse climatic conditions and range from good milch (of dairy merit) animals to extreme draught types. These breeds are known for their survival under inadequate feeding and low-input production practices naturally. Further, due to evolutionary divergence, *B. indicus* and *B. taurus* are expected to have variations in the candidate genes related to dairy traits. Keeping in view the scanty information available in Indian native cattle breeds, the present study was aimed to (i) sequence the full-length *αS1-CN5*′ in 13 Indian zebu breeds; (ii) search for putative TFs based on the indicine sequence (sequence specific to Indian zebu cattle); (iii) check if detected polymorphisms lie within identified TFBSs; (iv) identify variations within indicine breeds and their comparison with taurine breeds; and (v) identify homologies in the regulatory domains as well as phylogenetic relationship for *αS1-CN5*′ from different mammalian species.

## 2. Materials and Methods

### 2.1. Sample Collection and Isolation of Genomic DNA

For characterization of *αS1-CN5*′ and to determine the variants/haplotypes among Indian zebu cattle, blood samples of 19 unrelated animals from 13 breeds from diverse agroclimatic zones were collected from their respective native breeding tracts. The selected breeds and their respective sample sizes (given in parenthesis) represented dairy {Gir (1), Tharparkar (2), Rathi (2), Red Sindhi (2), and Sahiwal (2)}, draught {Amritmahal (1), Kangayam (1), and Red Kandhari (2)}, and dual purpose {Deoni (1), Gaolao (1), Hariana (2), Kankrej (1), and Mewati (1)}. Genomic DNA was isolated by enzymatic Proteinase-K digestion (Sigma Chemical Co. St. Louis, MO, USA) followed by standard phenol-chloroform extraction procedure [20]. The quality of isolated genomic DNA was analyzed on SafeView^TM^ (NBS Biologicals Ltd., England) stained 0.6% agarose gel and was quantified through NanoView (GE Healthcare, UK).

### 2.2. PCR Primers and Pmplification of *αS1-CN5′*

Primer pairs *αS1-CN*-F1 (5′-CCAATCCAGATATTGAACCTGC-3′) and *αS1*-*CN*-R1 (5′-ATAGGAAAGTACCAATACTTGC-3′) were used to amplify a fragment of 1,639 bp including promoter, exon-I, and intron-I region of *αS1-CN*. The primers were designed through PRIMER2 software using cow genomic sequence data available at ENSEMBL database (BTAU 4.0:6). PCR reaction was performed in 25 *μ*L reaction mixture containing 100-150 ng of genomic DNA, 5 p mole of each primer, 200 *μ*M of dNTPs mix, 1X PCR buffer, 1.5 mM MgCl_2,_ and 1.5 unit of *Taq* DNA polymerase (Invitrogen, Carlsbad, CA, USA) and was carried out in Mastercycler ep Gradient thermal cycler (Eppendorf, Germany) using thermal cycling conditions as initial denaturation at 95°C for 2 min, followed by 30 cycles at 95°C for 60 sec, 59°C for 45 sec and 72°C for 2.30 min with a final extension at 72°C for 10 min. The amplicons were electrophoresed through 1.2% SafeView stained agarose gel and were visualized under UV transilluminator. 

### 2.3. Sequencing, Annotation and Comparative Analysis of *αS1-CN5′*

The PCR product from each sample was purified using Exonuclaese I/Calf Intestinal Phosphatase (*Exo-CIP*) enzymatic treatment and used to sequence *αS1-CN5*′ region using forward primer *αS1-CN*-F1 and three additional internal primers (P1F: 5′-GTTCCTGTCATACAACTGTG-3′, P2F: 5′-ACTGGACACGACTTAGAAAC-3′, and P3F: 5′-CAATGCCATTCCATTTCCTG-3′) designed on the 1,639 bp amplicon. Sequencing reactions were performed with BigDye v3.1 cycle Sequencing Kit in an ABI PRISM 3130 Genetic Analyzer (Applied Biosystems, Foster City, CA, USA). The resulting sequences were aligned and polymorphic sites identified from sequence comparison were confirmed through manual inspection. The transcriptional factor binding sites were identified using TESS (http://www.cbil.upenn.edu/cgi-bin/tess/tess/), MATCH [21] (http://www.gene-regulation.com/cgi-bin/pub/programs/match/bin/match.cgi/), TRANSFAC [22] (http://www.biobase-international.com/), AliBaba2.1 Search engine [23] (http://www.gene-regulation.com/pub/programs/alibaba2/index.html), and from the literature [15, 19]. The potential functions of the putative TFBSs were determined from TRANSFAC database. PHASE v2.1.1 software [2426] was used to analyze identify haplotypes. The frequency of variations was calculated as number of animals with variation/total population size, whereas breed-wise frequency was estimated as number of mutations within a breed/total mutations. Linkage disequilibrium (LD) measures, $D^{\prime }$ and $r^{2}$, between all single nucleotide polymorphisms (SNPs) were estimated using Arlequin v3.5 software [27]. To determine the homologies among the DNA binding domain, sequences of *αS1-CN5*′ from major milk producing mammalian species (*B. indicus, B. taurus, B. bubalis, and C. hircus*) were extracted from GenBank and Ensemble databases. Molecular Evolutionary Genetic Analysis (MEGA) software version 5.0 [28] was used for the comparative sequence analysis and phylogenetic sequence analyses employing the Neighbor-Joining (NJ) method as this method does not require the assumption of a constant rate of evolution. Genetic distances were estimated by the p-distance model and standard errors of the estimates were obtained through 5,000 bootstrap replicates. 

## 3. Results

### 3.1. Sequence Analysis of Flanking Region of Alpha S1-Casein ( *αS1-CN5′*)

**Figure 1:**
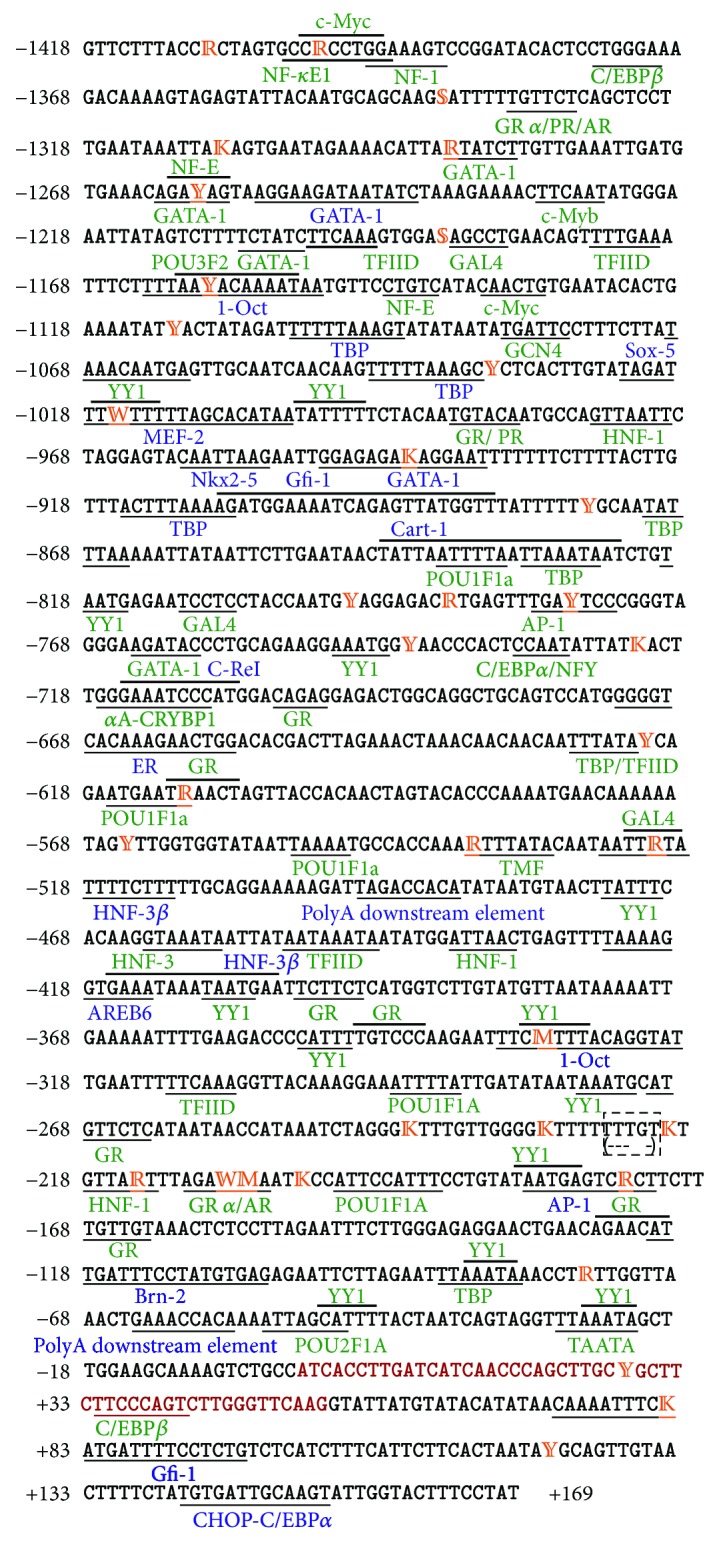
Promoter region of *alpha S1-casein* gene in Indian zebu cattle. Sites of variations are marked with IUPAC nucleotide codes, R: A/G, S: C/G, Y: C/T and K: G/T. Site of deletion among Indian cattle is represented in parenthesis. Region in bold nucleotides marks 5′UTR. Transcriptional start site is marked as +1. Abbreviations: AP: activator protein; AR: androgen receptor; AREB6: Atp1a1 regulatory element binding protein 6; CAAT: CAAT box; C/EBP: CCAAT/enhancer binding protein; Cart-1: cartilage homeoprotein 1; c-Myb: cartilage homeoprotein 1; c-ReI: nuclear factor kappa B c-Rel; ER: estrogen receptor; GATA-1: GATA-binding factor 1; Gfi-1: Zinc finger protein Gfi-1; GR: glucocorticoid receptor; HNF-1: hepatocyte nuclear factor; MEF-2: myocyte enhancer factor 2A; MGF (MPBF): mammary gland factor; NF: nuclear factor; Nkx2-5: homeobox protein Nkx-2.5; Oct-1: octamer-binding factor; POU1F1a: transcription factor 1 (Pit1, growth hormone factor 1); POU2F1a: POU domain, class 2, transcription factor 1; POU3F2: POU domain, class 3, transcription factor 2; PR: progesterone receptor; Sox-5: SRY-related HMG-box gene 5, Sp1: specificity protein 1; TBP: TATA-box-binding protein; TFIID: transcription factor IID, TATA-box-binding protein; TMF: TATA element modulatory factor; YY1: Yin Yang factor.

Sequencing the amplicon of 1,639 bp contig of 1,587 bp of *αS1-CN5*′ including 1418 bp of promoter region, 53 bp of exon-I, and 116 bp of intron-I region was analyzed in the present study. A total of 31 putative binding sites were identified within the promoter region (Figure 1, see supplementary Table S1 a,b in supplementary material available online at http://dx.doi.org/10.5402/2013/926025). Apart from consensus sequences of CAAT box and TATA box, the promoter region contained dense array of potential transcriptional factor binding domains as AP (activator protein), AR (androgen receptor), AREB6 (Atp1a1 Regulatory Element Binding protein 6), CAAT (CAAT box), C/EBP (CCAAT/enhancer binding protein), Cart-1 (cartilage homeoprotein 1), c-Myb (cartilage homeoprotein 1), c-ReI (Nuclear Factor kappa B) c-Rel, ER (estrogen receptor), GATA-1 (GATA-binding factor 1), Gfi-1 (Zinc finger protein Gfi-1), GR (Glucocorticoid receptor), HNF-1 (Hepatocyte Nuclear Factor), MEF-2 (myocyte enhancer factor 2A), MGF/MPBF (mammary gland factor), NF-E (Nuclear Factor-E), Nkx2-5 (Homeobox protein Nkx-2.5), Oct-1 (Octamer-binding factor), POU1F1a (transcription factor 1 (Pit1, growth hormone factor 1)), POU2F1a (POU domain, class 2, transcription factor 1), POU3F2 (POU domain, class 3, TF2), PR (progesterone receptor), Sox-5 (SRY-related HMG-box gene 5), Sp1 (Specificity Protein 1), TBP (TATA-box-binding protein), TFIID (Transcription factor IID), TMF (TATA element modulatory factor), and YY1 (Ying-Yang factor). Binding of most of the transcription factors to their respective sites was associated with basal, tissue specific developmental, and *cis-trans* gene regulation [2, 6, 7, 9]. All the identified sites showed high core match and matrix match similarity with a minimum value of 83.8%, 80.8% and a maximum value of 100% for each, respectively (Supplementary Table S1a). The conserved regulatory element, TATA box, was located between −22 and −28 bp while CAAT box was located between −52 and −57 upstream to transcriptional start site of *αS1-CN* gene (Figure 1). The position of TATA box in Indian zebu cattle was found to be consistent with that of other ruminants like *B. taurus*, *Capra hircus*, and *Ovis aries*.

### 3.2. Variation Analysis of *αS1-CN5′* among Indian Zebu Cattle. 

Comparative sequence analysis revealed a high mutation rate of 1 SNP/42 bp with presence of 39 variations (36 including four Indels within promoter region, 1 in exon-I and 2 in the intron-I region) observed within *αS1-CN5*′ in Indian zebu cattle breeds. Of the observed SNPs, 22 (56.41%) and 13 (43.59%) were transitions and transversions, respectively. The transition/transversion rate ratios were k1=3.062 (purines) and k2=4.386 (pyrimidines) while overall transition/transversion bias R was 1.585, with a total of 1587 positions in *αS1-CN5*′. Within promoter region, 36 nucleotide substitutions and 4 consecutive Indels (at −224 to −221 (TTGT>- - - -) with respect to transcriptional start site) were observed (Table 1). Throughout the region screened, variation at −722 (T > A) exhibited highest frequency (0.68) while 16 variations showed least frequency of 0.05 (Table 1). 

Breed-wise distribution of SNPs among the Indian zebu cattle breeds revealed Gir (dairy breed) to be most polymorphic with 62% (24/39) of the observed variations, whereas Gaolao (dual utility type) with 8% (3/39) variations was least polymorphic. Across different utility categories (diary, dual and draught), dairy breeds showing all the observed variations (100%; 39/39) were the most polymorphic followed by draught (54%; 21/39) and dual (33%; 13/39) purpose breeds. None of the variations were specific to breed utility category. Amongst Indian zebu cattle; 26 out of 39 variations (67%) observed within *αS1-CN5*′ were found to be novel as they have not been reported in any other cattle breeds (Table 1). For the observed variations, 27 haplotypes were predicted using software PHASE 2.1 (Supplementary Table S2). The majority (41%) of these haplotypes were confined to dairy animals (11/27), followed by dual (33%; 9/27) and draught (22%; 6/27) purpose animals. Amongst the observed haplotypes, a single haplotype (AS1_INC20) was shared in dairy, dual, and draught purpose breeds.

Among 36 variations observed in the promoter region; 15 were located within the putative TFBS influencing the binding affinity of their respective TFs, thus possibly affecting the gene transcript. Further, in intron-I, variation at position 82 (T > A) was also located within Gfi-1 TFBS (Table 1). Many of the observed variations influenced more than one nuclear factor (Table 1). However, observed deletions in *αS1-CN5*′ did not affect any known TFBS. Across the variations at DNA binding domains, −1259  (T > C) is located within the GATA-1 and NF-E exhibited maximum frequency (0.63).

### 3.3. Homologies of Regulatory Domains among Major Dairy Species

**Figure 2:**
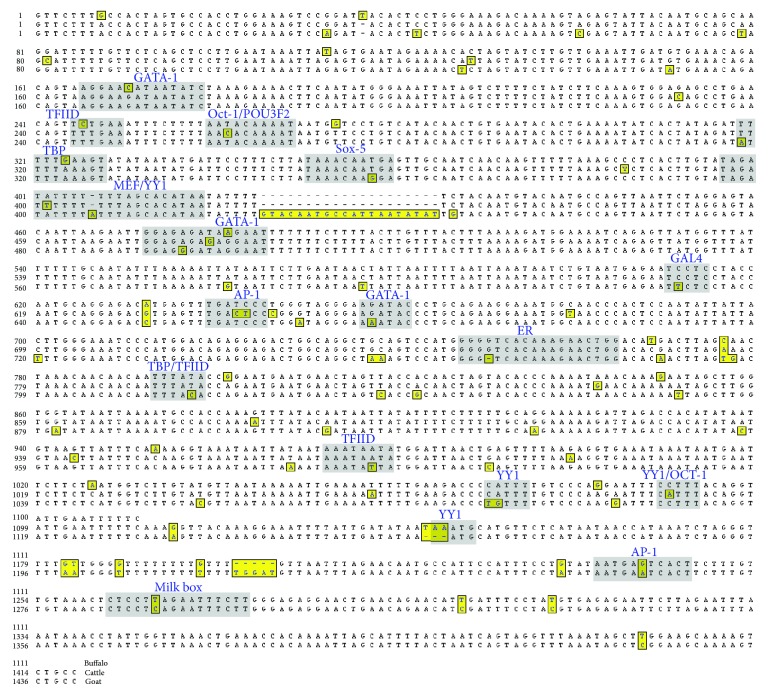
Homology between the nucleotide sequences of *αS1-CN5*′ for buffalo (upper lane), cattle (middle lane), and goat (lower lane). Variations are highlighted and marked in boxes, whereas gaps are represented by dashes. Only the putative TFBSs affected due to variations are marked in shaded regions.

Sequence comparison of *αS1-CN5*′ among major livestock species of dairy purpose (cattle, buffalo, and goat) revealed divergence at binding domain for several ubiquitous TFs and motifs specific to mammary gland and hormone receptors. Amongst the 31 different TFB elements annotated for Indian zebu cattle, 12 showed variations (Figure 2). These variable regions included transcriptional activators such as, ER, MEF, 16 bp milk box, and TBP, repressors of gene regulation such as YY1 and AP-1, while others were related with basal regulation of gene expression such as GAL4, GATA-1, Oct-1, POU3F2, Sox-5, and TFIID (Figure 2). 

### 3.4. Genetic Distance and Phylogeny among Different Mammalian Species

**Figure 3:**
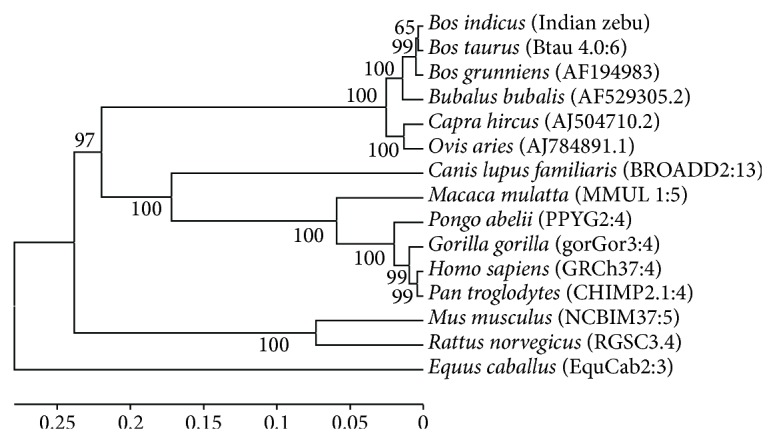
Evolutionary relationship of *alpha S1-casein* gene promoter region across different mammalian species. Databank accession numbers are given in the parenthesis.

Analysis of genetic distances at nucleotide level, using p-distance model based on pairwise deletion, revealed highest homology of Indian zebu cattle sequence with *B. taurus *(99.3%), followed by *B. grunniens* (99.1%), *Bubalus bubalis* (97.2%), *Ovis aries* (94.8%), *Capra hircus* (95.5%), *Canis lupus familiaris *(60%), *Gorilla gorilla* (54.3%), *Macaca mulatta* (54.2%), *Pongo abelii* (54.5%), *Homo sapiens* (54.8%), *Pan troglodytes* (54.7%), and *Rattus norvegicus* (48.6%). The analysis revealed Indian native cattle to be closest to *B. taurus* followed by yak and buffalo and most distant from *Equus caballus *(43.8%). Phylogenetic relationship based on UPGMA with 5,000 bootstrap replicates for *αS1-CN5*′ among 15 different mammalian species revealed four major groups. Ruminants from Bovidae family (cattle, yak, buffalo, goat, and sheep) were grouped together; members of Hominidae family (human, chimpanzee, orangutan, and gorilla) and monkey formed another group; rat and mouse from Muridae family were clustered together, while, horse from Equidae family was distinctly separated (Figure 3). 

## 4. Discussion

Due to close linkage of four casein genes, regulatory domains of one casein gene might influence the other caseins as well in addition to the respective casein [29]. It is pertinent to study variation in *αS1-CN* regulatory region as it is located at the 5′ end of casein group with orientation in the sense direction and most likely its 5′ region controls the transcription regulation of other caseins [10]. Further, compared to other caseins, *αS1-CN5*′ is the most variable [3] and these variations might influence the encoded transcripts and hence the milk composition and properties. Evidence for significant association of mutations within the regulatory region of casein complex with production traits across different taurine (*B. taurus*) breeds has been provided in number of studies [6, 7, 9, 10]. 

In the present study, sequence characterization of *αS1-CN5*′ among Indian zebu cattle (*B. indicus*) revealed a dense region of binding sites for tissue-specific factors, hormone receptors, and ubiquitary transcription factors with few overlapping binding sites. Overall 39 variations identified in Indian zebu cattle breed indicated high variability of *αS1-CN5*′. The polymorphic nature of *αS1-CN5*′ has also been reported by Schild and Geldermann [19] with 17 variable sites including 2 indels among 13 genetically heterogeneous groups of cows and Ibeagha-Awemu et al. [12] while analyzing nine *B. indicus* and three *B. taurus* breeds from Cameroon and Nigeria. Out of these 17 variations, 13 were similar to those observed for Indian zebu cattle in the present study, while 4 variations at positions −728 (− > T), −733 (T > C), −774 (C > T), and −820 (G > A) were unique to *B. taurus *(Table 1). Among the 4 variations unique to *B. taurus* the variation −728 (− > T) was genotyped using *Ssp*I restriction site and results suggested significant association of heterozygous genotype (− > T) with average higher *α*-S1 protein content in taurine breeds [6, 7, 9, 19, 30]. This variation at −728 (− > T) has been observed to have close linkage with −175  A > G (intragenic haplotype; [4]). Further, intergenic haplotypes have also been reported for *αS1-CN5*′ variation −728 (− > T) with variation in *αS1-CN5'* −1084 C > T and −186 T > C and *β* -*CN5*′ (−109 C > G) [7]. However, in contrast to such reports, variation/deletion was not observed at position −728 among the analyzed Indian cattle (*B. indicus*) breeds and all animals were homozygous for T allele (TT). Another important variation at −175 (A > G) located within the binding site of ubiquitously expressed AP-1 TF [19] showed different genotypic pattern across *B. taurus* and Indian native cattle breeds. The particular variation was genotyped across 62 Simmental and 80 German Holstein cows by Kuss et al. [13, 14] and reflected association of heterozygous AG genotype with high *α*S1-CN protein content. Conversely, none of the animals in our study showed heterozygous AG genotype at position −175, as all were either homozygous with GG or AA genotype. These findings clearly demonstrate the nucleotide divergence in the regulatory region of *αS1-CN5*′ across Indian and taurine cattle breeds.

Out of 39 variations observed in the present study, 16 were located within putative TFBS, some of which are ubiquitously expressed and involved in regulation of tissue-specific gene expression. The variations located within transcriptional activators included −1158 T > C and −330 A > C, positioned in the potential binding sites for ubiquitously expressed Oct-1 that could possibly change the transcriptional mechanism. The −1158 T > C also overlaps with POU3F2 TFBS specific to nervous system and binds Oct-1 (Figure 1, Supplementary Table S3). The variation at −1016 A > T was located within MEF-2 (regulator of cellular differentiation). Among the group of transcriptional repressors within the *cis*-acting elements, observed variation c536 A > G was positioned at TMF binding domain which represses activation of TATA box and 82 T > A (intornic region variation) within the Gfi1 TFBS (Supplementary Table S3). Some of the variations were located within the binding sites for TFs with activator and the repressor activity was; −1016 A > T and −330 A > C within ubiquitously distributed YY1 TFBS; −610 G > A, −207 A > T and −206 C > A within GR TFBS while; −778 C > T and −175 A > G within AP-1 TFBS. AP-1 is a known transcriptional activator, but few studies also suggest its role as repressor for *αS1-CN5*′ [13, 14].

The variations located within other important TFBS included −1399 A > G and −1259 T > C, located within Nuclear TFs (NF-Kappa E1 and NF-E, resp.) (Table 1) which are nuclear proteins with unknown specific function. −1399 A > G also overlaps with binding domain of c-Myc (Figure 1, Table 1). Similarly, variations −1288 G > A, −1259 T > C, and −941 T > G are marked within the GATA-1. Both c-Myc and GATA-1 are regulators involved in cell proliferation and cell growth. Under category of tissue-specific TFs, the observed variations were G-610A located within POU1F1a that influences secretion from pituitary gland and has *trans*-activation activity; −214 A > G within the liver specific activator, HNF-1 that acts in cooperation with other TFs. Although not tissue specific, variation at −521 A > G was sited within the TF GAL4 that mediates transactivation of gene regulation (Supplementary Table S3). Additionally, variations at −207 A > T and −206 C > A overlapping with the binding domain for GRalpha were located within AR that mediates androgen-specific gene regulation. Eleven out of the sixteen above-discussed variations occurring within important putative TFBSs are specific to Indian zebu cattle and have not been observed in any other breed>species. The remaining five variations (−1016 A > T, −53 A > G, −521 A > G, −330 A > C, and −175 A > G) are common with *B. taurus* (Table 1). The effect of these variations, individually or in combination, could influence the regulation of *αS1-CN *gene expression effectively. The variations from *B. taurus *counterpart at genomic level also indicate the possible differences in milk performance traits of the two subspecies. Also, homology differences of regulatory sequences among major dairy species (cattle, buffalo, and goat) might be responsible for difference at production level. As regulation of gene expression is under multifactorial control, there is a need to focus on haplotypes rather than individual variations.

The present study generates the knowledge related to variations in naturally evolved Indian cattle breeds within regulatory region of *αS1-CN *gene, wherein such information was lacking. The novel variations found in Indian cattle breeds may be responsible for differential content of milk components as compared to taurine breeds. This study needs to be extended further in combination with protein coding gene polymorphism (intragenic haplotypes) to evaluate effects of promoter polymorphism on milk production traits. The ability to link sequence variability to dairy traits in context of other members of casein family using SNP chip or other tools could be important. This would lead to efficient utilization of resources like Indian native cattle impacting the socioeconomic structure of large population in India. 

## Supplementary Material

Supplementary material contains information for identification of potential TFBSs within *αS1-CN5′* using MATCH and TESS software, list of haplotypes for *αS1-CN5′* among Indian cattle and predictive role of various putative regulatory domains affected due to polymorphism

## Figures and Tables

**Table 1:** Frequency of variations within the *αS1-CN5'* among Indian zebu cattle breeds in comparison with *B. taurus* (BTAU 4.0:6).

Sr. number	Region	Position	Variation	Allele	AMC	DEC	GAC	GIC	HAC	KJC	KYC	MEC	RAC	RKC	RSC	SAC	THC	Overall frequency (= *n/N*)	Potential TFBS	Novel
1	Promoter	-1408	A/G	G:	—	—	—	—	—	—	—	—	—	—	1	—	—	0.11	—	Yes
2	′′	-1399	A/G	G:	—	—	—	—	—	—	—	—	0.5	—	1	—	—	0.16	NF-kappaE1/c-Myc	Yes
3	′′	-1338	G/C	C:	1	—	—	1	—	—	1	—	—	—	—	0.5	—	0.21	—	Yes
4	′′	-1307	T/G	G:	1	—	—	1	—	—	1	—	—	—	—	0.5	—	0.21	—	Yes
5	′′	-1288	G/A	A:	—	—	—	1	—	—	—	—	—	—	—	—	—	0.05	GATA-1	Yes
6	′′	-1259	T/C	C:	1	1	—	1	—	1	1	1	0.5	1	0.5	0.5	0.5	0.63	GATA-1/NF-E	Yes
7	′′	-1188	C/G	G:	—	—	—	1	—	—	—	—	—	—	—	—	—	0.05	—	Yes
8	′′	-1158	T/C	C:	1	1	—	—	0.5	1	1	—	—	0.5	0.5	0.5	0.5	0.47	Oct-1/POU3F2	Yes
9	′′	-1111	C/T	T:	—	—	—	1	—	—	—	—	—	—	—	—	—	0.05	—	Yes
10	′′	-1034	T/C	C:	1	—	—	1	—	—	—	—	—	—	—	—	—	0.11	—	Yes
11	′′	-1016	A/T	T:	1	—	—	1	—	1	1	—	—	0.5	—	0.5	—	0.32	MEF-2/YY1	—
12	′′	-941	T/G	G:	1	—	—	—	—	1	1	—	—	—	—	—	—	0.16	GATA-1	Yes
13	′′	-876	C/T	T:	1	—	—	1	—	1	1	1	0.5	1	0.5	0.5	—	0.53	—	—
14	′′	-796	T/C	C:	1	—	—	1	—	1	1	—	0.5	0.5	0.5	0.5	0.5	0.47	—	—
15	′′	-788	G/A	A:	—	—	—	1	—	—	—	—	—	—	—	—	—	0.05	—	Yes
16	′′	-778	C/T	T:	—	—	—	1	—	—	—	—	—	—	—	—	—	0.05	AP-1	Yes
17	′′	-741	C/T	T:	1	—	—	—	—	1	1	—	—	—	—	—	—	0.16	—	—
18	′′	-722	T/A	A:	—	—	1	—	1	—	—	1	0.5	1	1	1	1	**0.68**	—	—
19	′′	-621	C/T	T:	—	1	1	—	1	—	—	—	—	1	1	1	1	0.63	—	—
20	′′	-610	G/A	A:	—	—	—	1	—	—	—	—	—	—	—	—	—	0.05	POU1F1a/GR	Yes
21	′′	-565	C/T	T:	—	—	—	1	—	—	—	—	—	—	—	—	—	0.05	—	Yes
22	′′	-536	A/G	G:	—	—	—	1	—	—	1	1	—	0.5	—	0.5	0.5	0.32	TMF	—
23	′′	-521	A/G	G:	—	—	—	—	—	1	1	—	—	0.5	0.5	—	—	0.21	GAL4	—
24	′′	-330	A/C	C:	—	1	—	1	—	1	1	1	0.5	0.5	0.5	0.5	0.5	0.53	YY1/Oct-1	—
25	′′	-241	T/G	G:	—	—	—	1	—	—	—	—	—	—	—	—	—	0.05	—	Yes
26	′′	-230	T/G	G:	—	—	—	1	—	—	—	—	—	—	—	—	—	0.05	—	—
27	′′	-224	T/(-)	(-):	—	—	—	—	—	—	1	—	0.5	—	—	0.5	0.5	0.21	—	Yes
28	′′	-223	T/(-)	(-):	—	—	—	—	—	—	1	—	0.5	—	—	0.5	0.5	0.21	—	Yes
29	′′	-222	G/(-)	(-):	—	—	—	—	—	—	—	—	0.5	—	—	—	—	0.05	—	Yes
30	′′	-221	T/(-)	(-):	—	—	—	—	—	—	—	—	0.5	—	—	—	—	0.05		Yes
31	′′	-214	A/G	G:	—	—	—	1	—	—	—	—	—	—	—	—	—	0.05	HNF-1	Yes
32	′′	-207	A/T	T:	—	—	—	1	—	—	—	—	—	—	—	—	—	0.05	GRalpha/AR	Yes
33	′′	-206	C/A	A:	—	—	—	1	—	—	—	—	—	—	—	—	—	0.05	GRalpha/AR	Yes
34	′′	-202	G/T	T:	—	—	—	1	—	—	—	—	—	—	—	—	—	0.05	—	Yes
35	′′	-175	A/G	G:	—	—	—	—	—	—	1	—	—	—	—	1	—	0.16	AP-1	—
36	′′	-76	G/A	A:	1	—	—	1	—	—	1	—	—	—	—	0.5	—	0.21	—	—
37	Exon-I	28	T/C	C:	1	—	—	—	—	—	1	—	—	—	—	0.5	—	0.16	—	—
38	Intron-I	82	T/A	A:	—	—	—	1	—	—	—	—	—	—	—	—	—	0.05	Gfi-1	Yes
39	′′	122	C/T	T:	—	1	1	—	1	—	—	1	—	0.5	0.5	—	0.5	0.42	—	Yes

Breed wise frequency:					0.31	0.13	**0.08**	**0.62**	0.1	0.23	0.44	0.15	0.26	0.28	0.28	0.41	0.26			

A	Promoter	$-820^{*}$	G/A	—																—
B	′′	$-774^{*}$	C/T	—																—
C	′′	$-733^{*}$	(-)/C	—																—
D	′′	$-728^{*}$	(-)/T	—																—

AMC: Amritmahal, DEC: Deoni, GAC: Gaolao, GIC: Gir, HAC: Haryana, KJC: Kankrej, KYC: Kangyam, MEC: Mewati, RAC: Rathi, RKC: Red Kandhari, RSC: Red Sindhi, SAC: Sahiwal, THC: Tharparkar, *n*: number of individuals with the variation, *N*: total number of animals studied.

∗Variations reported in *B. taurus* [19] but not observed in the present study.
