# Supplementary material for: Variations in the Regulatory Region of Alpha S1-Casein Milk Protein Gene among Tropically Adapted Indian Native (Bos Indicus) Cattle
Source: ISRN Biotechnol. 2013 Jan 14;2013:926025. doi: 10.5402/2013/926025 (PMC4393073; doi:10.5402/2013/926025)
Supplement: Supplementary file 1 — Supplementary material contains information for identification of potential TFBSs within αS1-CN5′ using MATCH and TESS software, list of haplotypes for αS1-CN5′ among Indian cattle and predictive role of various putative regulatory domains affected due to polymorphism [file 926025.f1.doc]

**Supplementary tables**:

**Supplementary table S1(a).** Identification of potential TFBSs within the *αS1-CN* *5’* through MATCH.

**Supplementary table S1(b).** Identification of potential TFBSs within *αS1-CN* *5’* through TESS.

**Supplementary table S2.** Haplotypes observed within *αS1-CN5’* among Indian zebu cattle.

**Supplementary table S3.** Predictive role of various putative regulatory domains affected due to polymorphism.

**Supplementary table S1(a):** Identification of potential TFBSs within the *αS1-CN* *5’* through MATCH

| Factor name | Position | Strand | Sequence motif* | Core match | Matrix match |
| --- | --- | --- | --- | --- | --- |
| GATA-1 | -1254 | (+) | aggaaGATAAtatc | 1 | 0.974 |
| 1-Oct | -1163 | (+) | tttaatACAAAataa | 0.838 | 0.808 |
| TATA | -1101 | (-) | tTTTTAaagt | 1 | 0.952 |
| Sox-5 | -1069 | (+) | taaACAATga | 1 | 0.997 |
| TATA | -1044 | (-) | tTTTTAaagc | 1 | 0.952 |
| MEF-2 | -1023 | (-) | tagattTATTTttagcacataa | 1 | 0.911 |
| Nkx2-5 | -960 | (-) | cAATTAag | 1 | 1 |
| GATA-1 | -948 | (+) | ggagAGATAggaat | 1 | 0.971 |
| TATA | -915 | (+) | acttTAAAAg | 1 | 0.955 |
| Gfi-1 | -907 | (+) | agatggaaAATCAgagttatggtt | 1 | 0.971 |
| Cart-1 | -843 | (-) | tattaattttAATTAaat | 1 | 0.981 |
| c-Rel | -716 | (-) | GAAAtccca | 1 | 0.988 |
| ER | -674 | (-) | ggGGTCAcaaagaactgg | 1 | 0.956 |
| HNF-3beta | -525 | (+) | aattaTATTTtcttt | 1 | 0.907 |
| PolyA downstream element | -495 | (-) | tagACCACa | 1 | 0.953 |
| AREB6 | -424 | (-) | taaaAGGTGaaa | 1 | 0.986 |
| HNF-3beta | -416 | (-) | gaaatAAATAatgaa | 1 | 0.953 |
| 1-Oct | -333 | (-) | ttcaTTTACaggtat | 0.909 | 0.838 |
| AP-1 | -182 | (-) | aatgAGTCAct | 1 | 0.996 |
| Brn-2 | -120 | (-) | attgATTTCctatgtg | 1 | 0.984 |
| PolyA downstream element | -64 | (-) | gaaACCACa | 1 | 0.957 |
| Gfi-1 | 73 | (-) | caaaatttctaTGATTttcctctg | 1 | 0.957 |
| CHOP-C/EBPalpha | 141 | (-) | tgtgaTTGCAagt | 1 | 0.919 |

*Nucleotides in upper case indicate the core sequence.

**Supplementary table S1(b):** Identification of potential TFBSs within *αS1-CN* *5’* through TESS

| **Factor** | **Begin** | **Sense** | **Sequence** | **La** | **La/** |
| --- | --- | --- | --- | --- | --- |
| NF-kappaE1 | -1402 | N | GCCACCTG | 16 | 2 |
| c-Myc | -1400 | N | CACCTG | 12 | 2 |
| NF-1 | -1395 | R | GGAAAG | 12 | 2 |
| C/EBPbeta | -1376 | N | CTGGRAA | 13 | 1.86 |
| GR alpha/ PR | -1332 | R | TGTTCT | 12 | 2 |
| AR | -1332 | N | TGTTCT | 12 | 2 |
| GATA-1 | -1288 | N | GTATCT | 12 | 2 |
| GATA-1 | -1262 | R | AGATAG | 12 | 2 |
| NF-E | -1261 | R | GATAG | 10 | 2 |
| c-Myb | -1230 | R | TTCAAT | 12 | 2 |
| GATA-1 | -1205 | R | TCTATC | 12 | 2 |
| TFIID | -1199 | N | TTCAAA | 12 | 2 |
| GAL4 | -1187 | N | AGCCT | 10 | 2 |
| TFIID | -1175 | R | TTTGAA | 12 | 2 |
| POU3F2 | -1160 | N | MATNNNWAAT | 12 | 1.2 |
| NF-E | -1143 | N | CTGTC | 10 | 2 |
| c-Myc | -1135 | R | CAACTG | 12 | 2 |
| GCN4 | -1083 | N | TGATTC | 12 | 2 |
| YY1 | -1017 | N | TATTT | 10 | 2 |
| YY1 | -1001 | N | TATTT | 10 | 2 |
| GR/ PR | -988 | R | TGTACA | 12 | 2 |
| HNF-1 | -976 | R | GTTAAT | 12 | 2 |
| TBP | -871 | N | TATTTAA | 14 | 2 |
| POU1F1a | -838 | R | ATTTTA | 12 | 2 |
| TBP | -831 | R | TTAAATA | 14 | 2 |
| YY1 | -819 | R | TAATG | 10 | 2 |
| GAL4 | -810 | R | TCCTC | 10 | 2 |
| AP-1 | -781 | R | TGACTCC | 14 | 2 |
| GATA-1 | -764 | R | AGATAC | 12 | 2 |
| YY1 | -747 | R | AAATG | 10 | 2 |
| C/EBPalpha / NF-Y | -732 | R | CCAAT | 10 | 2 |
| alphaA-CRYBP1 | -717 | N | GGGAAATCCC | 20 | 2 |
| GR | -702 | N | CAGAG | 10 | 2 |
| TBP/TFIID | -627 | R | TTTATA | 12 | 2 |
| POU1F1a | -616 | R | ATGAATG | 14 | 2 |
| GR | -611 | N | TGAACT | 12 | 2 |
| POU1F1a | -551 | N | TAAAAT | 12 | 2 |
| TMF | -536 | R | ATTTATA | 14 | 2 |
| GAL4 | -523 | R | TTATAT | 12 | 2 |
| YY1 | -474 | N | TATTT | 10 | 2 |
| HNF-3 | -463 | R | GTAAATA | 14 | 2 |
| TFIID | -451 | R | AATAAATA | 16 | 2 |
| HNF-1 | -437 | N | ATTAAC | 12 | 2 |
| YY1 | -408 | R | TAATG | 10 | 2 |
| GR | -400 | N | TCTTCT | 12 | 2 |
| YY1 | -350 | N | CATTT | 10 | 2 |
| GR | -345 | R | TGTCCC | 12 | 2 |
| YY1 | -331 | N | CATTT | 10 | 2 |
| TFIID | -311 | N | TTCAAA | 12 | 2 |
| POU1F1a | -292 | R | ATTTTA | 12 | 2 |
| YY1 | -276 | R | AAATG | 10 | 2 |
| GR | -270 | R | ATGTTCT | 14 | 2 |
| HNF-1 | -218 | R | GTTAAT | 12 | 2 |
| GR alpha | -210 | N | AGAACA | 12 | 2 |
| AR / GR | -210 | R | AGAACA | 12 | 2 |
| POU1F1a | -199 | N | ATTCCATT | 16 | 2 |
| YY1 | -184 | R | TAATG | 10 | 2 |
| GR | -168 | R | TGTTGT | 12 | 2 |
| GR | -125 | N | AGAACAT | 14 | 2 |
| TBP | -88 | R | TTAAATA | 14 | 2 |
| YY1 | -86 | R | AAATA | 10 | 2 |
| POU2F1a | -53 | N | ATTAGCAT | 16 | 2 |
| YY1 | -48 | N | CATTT | 10 | 2 |
| TBP | -28 | R | TTAAATA | 14 | 2 |
| YY1 | -26 | R | AAATA | 10 | 2 |
| C/EBPbeta | 34 | R | TTYCCAG | 13 | 1.86 |

Where, La: Log-likelihood score and La/: ( La / Length of the site

**Supplementary table S2:** Haplotypes observed within *αS1-CN5’* among Indian zebu cattle.

| Haplotype | Haplotypic sequences | Frequency | Breeds | Utility |
| --- | --- | --- | --- | --- |
| AS1_INC1 | AACGGCCCCCTGTCGCTTCGCAAATTAACGAACTC | 1.00 | Amritmahal | Draught |
| AS1_INC2 | AACGGCCCCTTGTCGCTTCGCAAATTAACGAACTC | 1.00 | Amritmahal | Draught |
| AS1_INC3 | AACGGCCCCTTGTCGCTTCGCGGCTTAACGGACTC | 2.00 | Kangayam | Draught |
| AS1_INC4 | AACGGCCCCTTTTCGCCATGCGACTTAACGGACTC | 2.00 | Sahiwal | Dairy |
| AS1_INC5 | AACGACGTTCTTTCATCTCATGACGGGTATAATAC | 2.00 | Gir | Dairy |
| AS1_INC6 | AAGTGCCCCTTGTCGCTTCGCAACTTAACGAGTTC | 1.00 | Kankrej | Dual |
| AS1_INC7 | AAGTGCCCCTTGTCGCTTCGCAGCTTAACGAGTTC | 1.00 | Kankrej | Dual |
| AS1_INC8 | AAGTGCCCCTATTCGCCATGCAACTTAACGAGTTC | 1.00 | Red Kandhari | Draught |
| AS1_INC9 | AAGTGCCCCTATCCGCCATGCGAATTAACGAGTTC | 1.00 | Tharparkar | Dairy |
| AS1_INC10 | AAGTGCCCCTATCTGCCTTGCAACTTAACGAGTTC | 1.00 | Deoni | Dual |
| AS1_INC11 | AAGTGCCTCTTTTTGCCATGCGGATTAACGAGTTC | 1.00 | Red Kandhari | Draught |
| AS1_INC12 | AAGTGCCTCTATTTGCCACGCGACTTAACGAGTTC | 1.00 | Mewati | Dual |
| AS1_INC13 | AAGTGTCCCTATCTGCCTTGCAACTTAACGAGTTT | 1.00 | Deoni | Dual |
| AS1_INC14 | AAGTGTCCCTATCTGCCATGCAAATTAACGAGTTC | 1.00 | Hariana | Dual |
| AS1_INC15 | AAGTGTCCCTATCTGCCATGCAAATTAACGAGTTT | 1.00 | Hariana | Dual |
| AS1_INC16 | AAGTGTCTCTATCCGCCATGCGAATTAACGAGTTC | 1.00 | Tharparkar | Dairy |
| AS1_INC17 | AAGTGTCTCTATCTGCCTCGCAAATTAACGAGTTC | 2.00 | Rathi | Dairy |
| AS1_INC18 | AAGTGTCTCTATCTGCCACGCGACTTAACGAGTTT | 1.00 | Mewati | Dual |
| AS1_INC19 | AAGTGTCTCTATCTGCCATGCAAATTAACGAGTTC | 2.00 | Gaolao, Hariana | Dual, dual |
| AS1_INC20 | AAGTGTCTCTATCTGCCATGCAAATTAACGAGTTT | 5.00 | Gaolao, Hariana, Red Kandhari, Tharparkar | Dual, dual, draught, dairy |
| AS1_INC21 | AAGTGTCTCTATCTGCCATGCAACTTAACGAGTTC | 1.00 | Red Kandhai | Draught |
| AS1_INC22 | AAGTGTCTCTATCTGCCATGCAACTTAACGGGTTC | 2.00 | Sahiwal | Dairy |
| AS1_INC23 | AGGTGCCTCTATTTGCCACGCAACTTAACGAGTTC | 1.00 | Rathi | Dairy |
| AS1_INC24 | AGGTGTCTCTATCCGCCACGCAACTTAACGAGTTC | 1.00 | Rathi | Dairy |
| AS1_INC25 | GGGTGCCCCTATTCGCCATGCAGCTTAACGAGTTC | 1.00 | Red Sindhi | Dairy |
| AS1_INC26 | GGGTGTCTCTATCTGCCATGCAAATTAACGAGTTT | 2.00 | Red Sindhi | Dairy |
| AS1_INC27 | GGGTGTCTCTATCTGCCATGCAGCTTAACGAGTTC | 1.00 | Red Sindhi | Dairy |

**Supplementary table S3.** Predictive role of various putative regulatory domains affected due to polymorphism

| **Transcription factors** | **Position** | **Predictive role** |
| --- | --- | --- |
| NF-kappaE1 (Nuclear factor E1) | -1395 to -1402 | - Nuclear factor |
| NF-E (Nuclear factor E) | -1257 to -1261 | - Nuclear factor |
| c-Myc | -1395 to -1400 | - Cell proliferation control - Post-transcriptional regulation of rRNA metabolism. |
| GATA-1 (GATA-binding factor 1) | -1284 to -1289, -1257 to -1262, -935 to -948 | - Regulated cell growth - Induces erythrocyte differentiation. |
| Oct-1 (octamer-binding factor) | -1149 to -1163, -319 to -333 | - Changes transcriptional mechanism - Influences other TFs ( Sp1, PR and/or GR) |
| POU3F2 (POU domain, class 3, transcription factor 2) | -1151 to -1160 | - Binds octamer DNA sequence and nervous system specific transcription factor N-Oct 3 (Brn-2) |
| MEF-2 (myocyte enhancer factor 2A) | -1002 to -1023 | - Regulators of cellular differentiation - Mediate stress response in some tissues. |
| AP-1 (activator protein-1) | -775 to -781, -173 to -183 | - Regulates gene expression - Controls a number of cellular processes including differentiation, proliferation, and apoptosis - Repressor role for *αS1-CN5’* |
| POU1F1a (Pituitary-specific positive transcription factor 1) | -610 to -616 | - Responsible for pituitary development and hormone expression |
| TMF (TATA element modulatory factor) | -530 to -536 | - Binds to TATA box and inhibit activation by TBP |
| GAL4 | -519 to -523 | - Mediates transactivation by trans-activating domains containing acidic amino acids |
| YY1 (Yin-Yang factor) | -1013 to -1017, -327 to -331 | - Represses and activates diverse number of promoters through histone modification. |
| HNF-1 (hepatocyte nuclear factor 1 homeobox A) | -213 to -218 | - Regulates hepatocyte specific gene expression. |
| GR/ GRalpha (Glucocorticoid receptor) | -606 to -611, --205 to -210 | - Activator or repressor in response to glucocorticoids. - Activates transcription of hormone-sensitive gene; while represses Oct-1. |
| AR (androgen receptor) | -205 to -210 | - Regulates androgen-dependent gene expression. |
| Gfi1 | +73 to +96 | - Transcriptional repressor - Role in stem cell differentiation and in granulocytic differentiation |
